# The Role of Aging Anxiety, Ageism, and Health Locus of Control on Middle-Aged Adults Health Outcomes and Behaviors

**DOI:** 10.1093/geroni/igab046.2318

**Published:** 2021-12-17

**Authors:** Hannah Bashian, Grace Caskie

**Affiliations:** 1 Lehigh University, Boston, Massachusetts, United States; 2 Lehigh University, Bethlehem, Pennsylvania, United States

## Abstract

Older adults with more ageist attitudes and aging anxiety and who endorse an external health locus of control (HLOC) have poorer mental and physical health and less engagement in healthy behaviors than those who report less ageist attitudes, aging anxiety, and endorse an internal HLOC. However, middle-aged adults have not been examined in this literature. Using Terror Management Theory as a framework, this study examined the relationship of middle-aged adults’ aging anxiety, ageist attitudes, and HLOC with health behaviors and mental and physical health outcomes. 391 middle-aged participants (40-55 years) completed measures of ageist attitudes, aging anxiety, HLOC (Internal, External, and Powerful Other), engagement in health behaviors, mental health, and physical health. The path analysis model demonstrated acceptable fit, χ2(2)=7.794, p=.02, CFI=.99, TLI=.92, RMSEA=.09). For health behaviors, eight of the 10 paths were significant; higher aging anxiety, higher ageist attitudes, and less endorsement of internal HLOC were related to less engagement in healthy behaviors. For mental health and physical health, five of the 10 paths were significant; in general, higher aging anxiety, higher ageist attitudes, and less endorsement of internal HLOC were related to poorer mental and physical health. This study demonstrated that middle-aged adults’ aging anxiety, ageist attitudes, and health locus of control are related to their health behaviors and mental and physical health. Furthermore, higher endorsement of specific forms of ageist attitudes and aging anxiety were related to worse reported mental and physical health and to less engagement in health behaviors. Implications of these findings will be discussed.

